# Intravenous, Intratracheal, and Intranasal Inoculation of Swine with SARS-CoV-2

**DOI:** 10.3390/v13081506

**Published:** 2021-07-30

**Authors:** Alexandra Buckley, Shollie Falkenberg, Mathias Martins, Melissa Laverack, Mitchell V. Palmer, Kelly Lager, Diego G. Diel

**Affiliations:** 1 Virus and Prion Research Unit, National Animal Disease Center, USDA, Agricultural Research Service, 1920 Dayton Avenue, P.O. Box 70, Ames, IA 50010, USA; Kelly.lager@usda.gov; 2 Ruminant Disease and Immunology Research Unit, National Animal Disease Center, USDA, Agricultural Research Service, 1920 Dayton Avenue, P.O. Box 70, Ames, IA 50010, USA; Shollie.falkenberg@usda.gov; 3 Animal Health Diagnostic Center, Department of Population Medicine and Diagnostic Sciences, College of Veterinary Medicine, Cornell University, 240 Farrier Rd, Ithaca, NY 14853, USA; mm3245@cornell.edu (M.M.); mp75@cornell.edu (M.L.); 4 Infectious Bacterial Diseases Research Unit, National Animal Disease Center, USDA, Agricultural Research Service, 1920 Dayton Avenue, P.O. Box 70, Ames, IA 50010, USA; Mitchell.palmer@usda.gov

**Keywords:** SARS-CoV-2, swine, inoculation

## Abstract

Since the emergence of severe acute respiratory syndrome coronavirus 2 (SARS-CoV-2), the susceptibility of animals and their potential to act as reservoirs or intermediate hosts for the virus has been of significant interest. Pigs are susceptible to multiple coronaviruses and have been used as an animal model for other human infectious diseases. Research groups have experimentally challenged swine with human SARS-CoV-2 isolates with results suggesting limited to no viral replication. For this study, a SARS-CoV-2 isolate obtained from a tiger which is identical to human SARS-CoV-2 isolates detected in New York City and contains the D614G S mutation was utilized for inoculation. Pigs were challenged via intravenous, intratracheal, or intranasal routes of inoculation (*n* = 4/route). No pigs developed clinical signs, but at least one pig in each group had one or more PCR positive nasal/oral swabs or rectal swabs after inoculation. All pigs in the intravenous group developed a transient neutralizing antibody titer, but only three other challenged pigs developed titers greater than 1:8. No gross or histologic changes were observed in tissue samples collected at necropsy. In addition, no PCR positive samples were positive by virus isolation. Inoculated animals were unable to transmit virus to naïve contact animals. The data from this experiment as well as from other laboratories supports that swine are not likely to play a role in the epidemiology and spread of SARS-CoV-2.

## 1. Introduction

Severe acute respiratory syndrome coronavirus 2 (SARS-CoV-2) emerged in China at the end of 2019 and has subsequently spread and infected humans around the globe [1,2]. Bats are likely the reservoir host for SARS-CoV-2 and bat coronaviruses showed sequence similarity to SARS-CoV-2 [3]. Historically, other zoonotic coronaviruses have been transferred to humans to through intermediate animal hosts, including masked palm civets for severe acute respiratory syndrome coronavirus (SARS-CoV) [4] and camels for Middle East respiratory syndrome coronavirus (MERS-CoV) [5]. Given the epidemiologic association of early reported cases of SARS-CoV-2 with a seafood and wildlife animal market in Wuhan, China in December 2019, identification of susceptible animal species that could serve as intermediate hosts or become reservoirs for the virus is a research priority [6].

Swine have a history of zoonotic transmission of viruses to humans including influenza A virus [7] and Nipah virus, for which fruit bats were identified as the natural reservoir host [8]. A HKU2-related bat coronavirus associated with swine acute diarrhea syndrome coronavirus (SADS-CoV) was recently discovered in 2016 and caused fatal disease outbreaks in swine in China near the geographic origin of the SARS-CoV outbreak [9]. In addition, swine are the natural host for other coronaviruses including porcine epidemic diarrhea virus, porcine deltacoronavirus, and porcine hemagglutinating encephalomyelitis virus [10,11].

SARS-CoV-2 is a single-stranded, positive-sense RNA virus that is a member of the genus *Betacoronavirus* in the subgenus *Sarbecovirus* [12]. Binding of the virus to a cellular receptor and internalization into the cell is critical for viral replication and a productive infection. The receptor for SARS-CoV-2 is angiotensin converting enzyme II (ACE2), which is also the receptor for SARS-CoV [13]. Phylogenetic and structural analyses of the ACE2 of multiple animal species predicted the binding propensity of multiple species’ ACE2 receptors to the receptor binding domain (RBD) on the spike protein of SARS-CoV-2. Predictive analysis suggested pig ACE2 binding abilities ranging from favorable to low [14,15,16].

Zhou et al. conducted infectivity studies with HeLa cells expressing swine ACE2 confirming SARS-CoV-2 was able to use the swine ACE2 protein for cellular entry [13]. Additional studies analyzed the location of the ACE2 receptor in swine tissues. The kidney was the tissue with the highest relative expression of ACE2, while respiratory tissues including lung, trachea, and nasal turbinates had very low levels of ACE2 mRNA [16]. Further in vitro analysis reported that SARS-CoV-2 is able to replicate in two swine cell lines, swine testicular cells and porcine kidney (PK-15) cells [17]. Interestingly, PK-15 cells were previously shown to support SARS-CoV replication [18]. These observations in cell culture in vitro suggested that swine could potentially be susceptible to SARS-CoV-2 infection.

Previous studies in which swine have been inoculated with SARS-CoV-2, have suggested that swine were not susceptible to SARS-CoV-2 infection due to lack of clinical signs, PCR detection in collected samples, and antibody response [17,19,20,21]. However, a recent study reported RT-PCR detection of SARS-CoV-2 in two nasal wash samples and one oral fluid sample, with the virus being isolated from a lymph node on 13 days post-inoculation (dpi) in pigs following intranasal inoculation [22]. In addition, another study reported seroconversion in swine challenged intramuscularly or intravenously [21]. Considering the number of pigs raised around the world and the extensive contact with humans, it is important to understand whether swine may play a role in the maintenance and spread of SARS-CoV-2.

In the present study, we assessed the susceptibility and potential transmission of SARS-CoV-2 in pigs, following three routes of inoculation: intravenous (IV), intratracheal (IT), and intranasal (IN). The IV inoculation route was of interest due to the levels of ACE2 receptor expressed in the kidneys of swine. The SARS-CoV-2 isolate used in our study (TGR1/NY/20) was obtained from a tiger that developed respiratory signs with evidence of transmission from a zookeeper [23]. This isolate is identical to other human SARS-CoV-2 isolates detected in New York City at the time of the outbreak and it contains the D614G mutation in the S protein.

## 2. Materials and Methods

### 2.1. Cells and Virus

Vero cells (ATCC^®^ CCL-81^™^) and Vero E6/TMPRSS2 (Japanese Cancer Research Resources (JCRB) Cell Bank, JCRB1819) were cultured in Dulbecco’s modified eagle medium (DMEM) supplemented with 10% fetal bovine serum (FBS), l-glutamine (2 mM), penicillin (100 U/mL), streptomycin (100 μg/mL) and gentamycin (50 μg/mL) and maintained at 37 °C with 5% CO_2_. SARS-CoV-2 was isolated on Vero CCL-81 cells from respiratory secretions from a Malayan tiger that developed respiratory signs (TGR/NY/20, GenBank accession number MT704317) [23,24,25]. Virus stock (passage 4) was clarified by centrifugation (1666× *g* for 10 min) and stored at −80 °C. A viral suspension containing 6.8 × 10^6^ tissue culture infectious dose 50 per ml (TCID_50_/_mL_) as determined by the Spearman-Karber method was used for swine inoculation.

### 2.2. Animal Experiment

All animal work was performed in accordance with an animal care and use protocol (ACUP ARS-2020-861) approved by the Institutional Animal Care and Use Committee at the National Animal Disease Center (NADC). Twenty-four 3-week-old pigs were purchased from a commercial swine herd (Wisconsin, USA) and transported to the NADC. Control pigs were housed in one Animal Biosafety Level 2 (ABSL-2) room, while challenged pigs were housed in separated pens in one Biosafety Level 3 Agriculture (BSL-3AG) room. For the challenge study, three routes were performed: IV (*n* = 4), IT (*n* = 4), and IN (*n* = 4). Sham cell culture lysate challenge was administered to control pigs (*n* = 2/route) with 2 control pigs untreated (*n* = 8 total). The IV group received 2 mL of inoculum injected into the jugular vein. The IT group was sedated using an intramuscular injection of ketamine (8 mg/kg of body weight; Pheonix, St. Joseph, MO. USA), xylazine (4 mg/kg; Lloyd Inc, Shenandoah, IA, USA), and Telazol^®^ (6 mg/kg; Zoetis Animal Health, Florham Park, NJ, USA) cocktail and 5 mL of inoculum was administered IT using a catheter and laryngoscope. Finally, an atomization device (LMA^®^ MAD Nasal™, Teleflex; Morrisville, NC, USA) was used for delivery of about 2.5 mL of the inoculum into each nostril for a total of 5 mL in the IN group.

To assess transmission, one contact pig was added to each challenge group and the control room on 2 dpi (*n* = 4 total). Temperatures were recorded daily using a subcutaneous microchip (LifeChip^®^ with BioThermo^®^ Technology, Destron Fearing, DFW Airport, TX, USA). Pigs were observed daily for clinical signs of infection including coughing, nasal discharge, and diarrhea. Nasal/oral and rectal swabs (Puritan Medical Products, Guilford, ME, USA) were collected from all animals on 0–7, 10, 12, 14, 18 and 21 dpi and placed individually in sterile tubes containing 2 mL of viral transport media (MEM with 1000 U/mL of penicillin, 1000 μg/mL of streptomycin, and 2.5 μg/mL of amphotericin B). Group oral fluids were collected from each pen with white cotton ropes on 4–7, 10, 12, 14, and 21 dpi. Wet portions of the rope were placed in a plastic bag and manually wrung out. Fluid from the bag was poured into 15-mL conical centrifuge tubes. Blood was collected via jugular venipuncture using serum separation tubes and EDTA tubes at 0, 3, 7, 14, and 21 dpi from all animals. Serum and buffy coat were harvested after centrifugation (1200× *g* for 25 min) of serum separator and EDTA tubes, respectively. All samples were stored in a −80 °C freezer until testing.

Following necropsy at 21 dpi, tracheal wash, lung lavage, and tissues (turbinates, tonsil, heart, kidney, liver, spleen, trachea, bronchi, lung, ileum, jejunum, colon, retropharyngeal lymph node (LN), mandibular LN, mesenteric LN, tracheobronchial LN, mediastinal LN, thymus, cerebellum, cerebrum, olfactory bulb) were collected. Samples were individually bagged, placed on dry ice, and transferred to a −80 °C freezer until testing. Additional tissue samples were collected and processed for standard microscopic examination and in situ hybridization (ISH). For this, tissue fragments of approximately ≤0.5 cm in width were fixed by immersion in 10% neutral buffered formalin (≥20 volumes fixative to 1 volume tissue) for approximately 24 h, and then transferred to 70% ethanol, followed by standard paraffin embedding techniques. Slides for standard microscopic examination were stained with hematoxylin and eosin (HE).

### 2.3. Nucleic Acid Extraction and Real-Time Reverse Transcriptase PCR (rRT-PCR)

Nucleic acid extraction and rRT-PCR was performed at the Cornell Animal Health Diagnostic Center (AHDC) as previously described [24,25]. Prior to extraction from tissues, 0.5 g of select tissues were minced, resuspended in 5 mL of DMEM, homogenized with a stomacher (Stomacher^®^ 80 Biomaster, one speed cycle of 60 s) and centrifuged (1966× *g* for 10 min). Briefly, an aliquot of 200 µL was used for RNA extraction from nasal/oral swabs, rectal swabs, buffy coat, serum, oral fluids, tracheal wash, lung lavage, and select tissues after thawing. The real-time RT-PCR was performed using the EZ-SARS-CoV-2 Real-Time RT-PCR assay (Tertacore Inc., Rockville, MD, USA). Real-time RT-PCR was performed on an ABI 7500 Fast instrument (Life Technologies, Carlsbad, CA, USA) run in standard mode with the following conditions: 1 cycle at 48 °C for 15 min, followed by 1 cycle at 95 °C for 2 min, and 45 cycles of 95 °C for 10 s and 60 °C for 40 s. Samples with Ct values less than 40 were considered positive.

### 2.4. Virus Isolation

Samples that tested positive for SARS-CoV-2 by rRT-PCR assay were subjected to virus isolation under biosafety level 3 (BSL-3) conditions at Cornell AHDC as previously described [23,24]. Briefly, 24-well plates of Vero E6/TMPRSS2 cells were inoculated with 150 µL of each sample for 1 h at 37 °C and replaced with DMEM supplemented with 10% FBS, L-glutamine (2 mM), penicillin (100 U/mL), streptomycin (100 μg/mL) and gentamycin (50 μg/mL). Plates were incubated at 37 °C with 5% CO_2_ monitored daily for cytopathic effect (CPE) for 3 days. If no CPE was visualized, those wells were subjected to two additional blind passages. At the end of the third passage subjected to an immunofluorescence assay (IFA) using a monoclonal antibody anti-SARS-CoV-2 nucleoprotein (clone B6G11) from Dr. Diel’s laboratory, goat anti-mouse IgG secondary antibody, and visualized under a fluorescence microscope.

### 2.5. Serological Analysis

Neutralizing antibodies against SARS-CoV-2 were measured using a virus neutralization (VN) assay performed under BSL-3 conditions at Cornell AHDC [24,25]. Briefly, Two-fold serial dilutions (1:8 to 1:4096) of serum samples were incubated with 100–200 TCID_50_ of SARS-CoV-2 isolate TGR/NY/20 for 1 h at 37 °C. Following incubation, 50 μL of a cell suspension of Vero cells was added to each well of a 96-well plate and incubated at 37 °C with 5% CO_2_ and for 48 h. The cells were fixed, permeabilized and subjected to IFA. Virus infectivity was assessed under a fluorescence microscope. Neutralizing antibody titers were expressed as the reciprocal of the highest dilution of serum that completely inhibited SARS-CoV-2 infection/replication.

### 2.6. In Situ Hybridization (ISH)

Paraffin-embedded tissues were sectioned at 5 μm and subjected to ISH using the RNAscope ZZ probe technology (Advanced Cell Diagnostics, Newark, CA, USA). In situ hybridization was performed to detect tissue distribution of SARS-CoV-2 nucleic acid in rRT-PCR positive tissues and matching control tissues using the RNAscope 2.5 HD Reagents–RED kit (Advanced Cell Diagnostics, Newark, CA, USA) as previously described [24].

ISH was also performed on the turbinates, tonsil, lung, and kidney to detect tissue distribution of mRNA ACE2 receptor distribution using the BaseScope 2.5 HD Reagents–RED kit (Advanced Cell Diagnostics, Newark, CA, USA) as previously described [24]. Proprietary ZZ probes targeting the region spanning AA 31–82 for the ACE2 receptor specific to *Sus scrofa* (BA-Ss-ACE2-1zz-st probe, ref# 900391) designed and manufactured by Advance Cell Diagnostics. The slides were counterstained with hematoxylin and examined by light microscopy using a Nikon Eclipse Ci microscope. Digital images were captured using a Nikon DE-Ri2 camera.

## 3. Results

### 3.1. Clinical Signs

**Figure 1:**
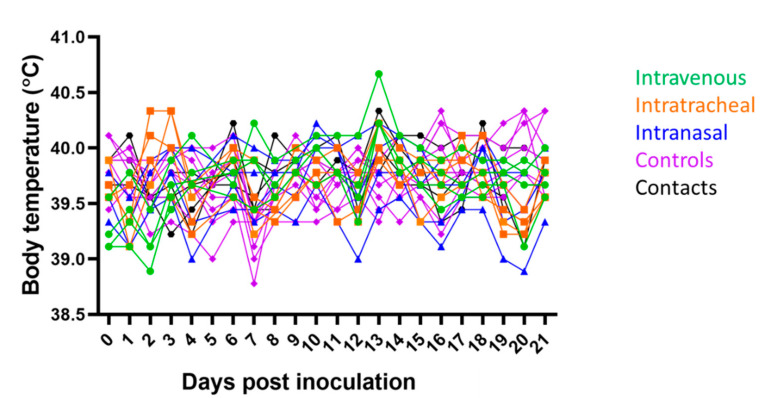
Daily pig body temperatures in degrees Celsius. Pigs inoculated intravenously are colored green, intratracheally orange, intranasally blue, controls purple, and contact pigs black.

Three-week-old pigs were challenged IV, IT, or IN to determine the susceptibility of swine to SARS-CoV-2. Immediately after IV challenge, pigs that received either virus or sham cell culture lysate began to vomit and shake but recovered within ten minutes. Otherwise, no other clinical manifestations were observed throughout the study in any of the challenge, control, or contact animals. Two IT challenged pigs had increased temperatures 2–3 dpi (40.3 °C), but only one pig (344) in the IV group had a temperature greater than 40.5 °C, which occurred on 13 dpi (Figure 1).

### 3.2. Nucleic Acid Detection of SARS-CoV-2 and Neutralizing Antibody Response

All SARS-CoV-2 challenged animals were negative by rRT-PCR and seronegative by VN assay on 0 dpi samples. All control animals were rRT-PCR negative for SARS-CoV-2 in selected samples and did not seroconvert. In addition, all samples collected from contact animals were rRT-PCR negative and there was no evidence of seroconversion.

The IV group had two pigs (344, 347) rRT-PCR positive on 3 dpi in the buffy coat and one pig (345) positive in a nasal/oral swab on 4 dpi (Table 1). The remaining samples were all negative by rRT-PCR. All pigs in the IV group developed VN titers between 32 and 64 on 7 dpi but a reduction in those titers was observed on 14 dpi (8 to 32, Table 2). By 21 dpi all pigs had a titer of 8, which was similar to pre-challenge titers.

In the IT group, all pigs were positive in nasal/oral swabs on 1 dpi (Table 1). On 2 dpi, SARS-CoV-2 RNA was detected in the nasal/oral swabs of only two pigs (350, 351). Beyond 2 dpi, only one pig (351) was rRT-PCR positive on 7 dpi. In rectal swabs, pig 350 was positive on 1, 2, and 7 dpi. Pig 351 had positive rectal swabs on 1 and 3 dpi. In addition, the IT group had rRT-PCR positive oral fluids on 6 dpi. Pig 351 had titers of 16 on 14 dpi and 8 on 21 dpi, while pig 350 had a titer of 8 at 14 dpi and developed a titer of 32 on 21 dpi (Table 2).

All animals in the IN group were positive in nasal/oral swabs on 1 dpi (Table 1). Two animals (353, 355) were rRT-PCR positive on 2 dpi and one animal (354) on 3 dpi. In rectal swabs, pig 355 had positive swabs on 1 and 2 dpi. Pig 352 had positive rectal swabs on 2, 3, 4, 5, 10, and 12. In addition, this group was rRT-PCR positive in oral fluids on 4 dpi. Only pig 352 showed evidence of a neutralizing antibody response with a titer of 32 on 14 dpi and 16 on 21 dpi (Table 2).

Select tissues (nasal turbinates, tonsil, mandibular LN, tracheobronchial LN, and lung) from challenged and contact pigs were chosen for rRT-PCR based on tissues that have been PCR positive in other published studies with swine and other susceptible species [20,22,24]. Some tonsil and LN tissues were rRT-PCR positive from at least one pig in each challenge group and in a contact pig in the IV group, but Ct values were relatively high (Table 3). All rRT-PCR positive samples including buffy coat, swabs, oral fluids, and tissues were subjected to virus isolation. All samples were virus isolation negative after three blind passages indicating a lack of infectious virus.

### 3.3. Histology and In Situ Hybridization

At necropsy, there were no lesions visible in any of the challenged, contact, or control animals. There were no histologic lesions present in any of the tissues collected at necropsy. RNAScope was performed on tissues that were rRT-PCR positive and matching control tissues and no staining to indicate the presence of SARS-CoV-2 RNA was observed in those tissues at 21 dpi.

**Figure 2:**
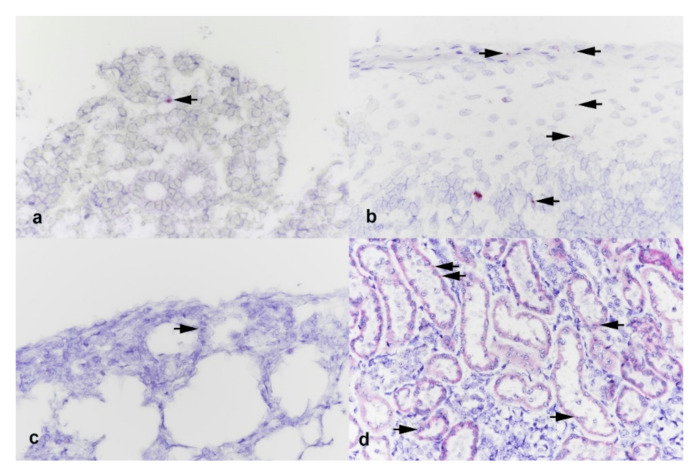
Photomicrographs of (**a**) turbinate, (**b**) tonsil, (**c**) lung, and (**d**) kidney from piglets experimentally inoculated with SARS-CoV-2. In turbinates rare labeling of ACE2 RNA was seen associated with submucosal glands (arrow). Labeling in the tonsil was visible in cells of various layers of the stratified squamous epithelium (arrows). While rare ACE2 RNA expression was noted in pulmonary interstitium (arrow), more moderate expression was noted in renal tubular epithelial cells (arrows). BaseScope ISH ACE2. Magnification = 40×.

Transcription of ACE2 was assessed in the turbinate, tonsil, lung, and kidney from 2 piglets from each challenge group by BaseScope (labeling of ACE2 mRNA). In turbinates rare labeling of ACE2 RNA was seen associated with submucosal glands and labeling in the tonsil was visible in cells of various layers of the stratified squamous epithelium (Figure 2). While rare ACE2 RNA expression was noted in pulmonary interstitium of the lung, more moderate expression was noted in renal tubular epithelial cells of the kidney (Figure 2).

## 4. Discussion

Although most swine SARS-CoV-2 challenge studies have previously reported that pigs challenged intranasally, intratracheally, intramuscularly, and/or intravenously were not susceptible to infection with SARS-CoV-2 [17,19,20,21]; Canadian researchers reported that pigs challenged intranasally were susceptible to SARS-CoV-2 [22]. We report rRT-PCR positive nasal/oral swabs and rectal swabs and VN activity in some animals from all challenge groups, however, viral nucleic acid detection and antibody titers were not sustained and there was no evidence of replicating virus via virus isolation.

There have now been several species that have been identified as being susceptible to experimental SARS-CoV-2 infection including hamsters [26,27], ferrets [19,20,28], cats [19], non-human primates [29,30], bats [20], and white-tailed deer [24]. In those studies, inoculated animals typically presented sustained viral replication and shed virus for about a week after inoculation; in addition, transmission to naïve contacts was demonstrated. In contrast to these susceptible species, pigs in this study only had intermittent rRT-PCR positive samples during the first week. Importantly, these samples were virus isolation negative and there was no evidence of transmission to naïve contacts, which is similar to results from challenge studies performed with another livestock species, cattle [25,31]. When the results from this study are compared to other animal species that have been determined to be susceptible to SARS-CoV-2, the lack of sustained replication and antibody response is evident.

The inoculum virus isolate used in this study (TGR1/NY/20) was originally obtained from a tiger from the Bronx zoo presenting respiratory signs and is identical to a virus recovered from a zookeeper; therefore, demonstrating its ability to jump from humans to animals [23]. This particular virus isolate contains the D614G mutation in the S gene, which was emerging in the US at the time of the outbreak in the animals and became the dominant genotype worldwide. It is important to note that the genome sequence of the TGR1/NY/20 isolate is identical not only to the sequence obtained directly from the clinical sample from the index Tiger 1 and its keeper, but also to several other human SARS-CoV-2 isolates detected in NYC at the time of the outbreak in the felids in the zoo [23]. Additionally, the same SARS-CoV-2 isolate was used in companion studies with cattle and white-tailed deer, which showed no productive infection in cattle, while deer were successfully infected and were able to transmit the virus to naïve contacts [24,25].

We have evaluated the susceptibility of pigs to SARS-CoV-2 following different inoculation routes (intranasal, intratracheal and intravenous). Results obtained following inoculation with all three routes were similar, with no evidence of productive virus infection being observed in any inoculated animal. Although the IV route is not likely a natural route of exposure, this route of inoculation was chosen based on previous work suggesting the highest levels of ACE2 RNA in swine were located in the kidney, while lower expression levels were observed in the respiratory tract [16,32]. Similar to previous reports, the highest levels of ACE2 expression in this study was found in the kidney.

Detection of SARS-CoV-2 RNA in secretions at early time points post-infection suggest likely detection of residual inoculum virus. Most rRT-PCR positive swab samples occurred during the first three days after infection. Although this timing does not completely rule out potential virus replication in the pig, the low viral loads as evidenced by high rRT-PCR Ct values suggests likely detection of inoculum virus. This is supported by the only positive buffy coat samples occurring on 3 dpi in the IV group and not in either the IT or IN group. In addition, there were a high number of rRT-PCR positive nasal/oral swabs in both the IN and the IT group on 1 and 2 dpi that would be consistent with the route of inoculation, which was not observed in the IV group. Beyond 1 dpi, all samples tested by PCR had Ct values greater than 31 indicating low levels of viral nucleic acid. This was further supported by the lack of virus isolation from rRT-PCR positive samples and lack of transmission to naïve contact animals.

Some pigs in this study developed a transient neutralizing antibody titer after inoculation. This was most clearly observed in the IV group at 7 dpi while those animals returned to pre-challenge titers by 21 dpi. A similar minimal transient neutralizing antibody response was reported in young calves challenged IT with SARS-CoV-2 [25]. The magnitude of neutralizing antibody response in humans after SARS-CoV-2 infection has been correlated with disease severity [33,34]. Humans with mild SARS-CoV-2 infections have shown transient to even absent immune responses which has been hypothesized to be correlated with lower levels of virus replication [35]. Interestingly, pigs with the most rRT-PCR positive samples in the IT and IN groups were the ones that developed some evidence of a neutralizing antibody response. Pigs in this study did not develop any clinical signs after inoculation. Researchers from Spain also reported antibody detection in pigs challenged IM and IV with SARS-CoV-2, but not in pigs challenged IT [21]. We hypothesize the lack of productive infection in swine contributed to the transient or lack of neutralizing antibody response observed in this study. The transient antibody responses detected are likely a result of the immune response to the viral antigen delivered in the inoculum.

Similar to other challenge studies in swine, there were no gross or histologic changes observed in necropsy tissues at 21 dpi [17,22]. One study reported a submandibular lymph node that was virus isolation positive at 13 dpi [22], while another tested tissues on 1 and 2 dpi and only found the trachea PCR positive from a single animal on 1 dpi [21]. Due to those reports and others from susceptible species, we focused viral detection efforts on lymph nodes, tonsil, and the respiratory tract. Although there were some PCR positive samples with high Ct values, they were virus isolation negative and there was no evidence of viral RNA using ISH.

This study reinforces the importance of performing in vivo work to confirm or reject in silico predictions of species susceptibility to SARS-CoV-2. Although, the SARS-CoV-2 S protein efficiently binds to swine ACE2 receptor and the virus is internalized and replicates in cell culture, animal challenge studies with various SARS-CoV-2 isolates have demonstrated that swine do not have sustained replication of SARS-CoV-2 and do not transmit virus to naïve contacts. Thus, it is unlikely that swine are a reservoir or contributing to the epidemiology and spread of SARS-CoV-2 in the human population. Continued research to determine animal species that could serve as reservoirs for the virus or play a role in transmission will remain important.

## Figures and Tables

**Table 1:** Summary of positive sample type and day as determined by rRT-PCR.

Group	Intravenous				Intratracheal				Intranasal			
Sample	N/O	R	BC	OF	N/O	R	BC	OF	N/O	R	BC	OF
DPI												
0	0/4	0/4	0/4	NS	0/4	0/4	0/4	NS	0/4	0/4	0/4	NS
1	0/4	0/4	NS	NS	4/4	2/4	NS	NS	4/4	3/4	NS	NS
2	0/4	0/4	NS	NS	2/4	2/4	NS	NS	2/4	2/4	NS	NS
3	0/4	0/4	2/4	NS	0/4	1/4	0/4	NS	1/4	1/4	0/4	NS
4	1/4	0/4	NS	0/1	0/4	0/4	NS	0/1	0/4	1/4	NS	1/1
5	0/4	0/4	NS	0/1	0/4	0/4	NS	0/1	0/4	1/4	NS	0/1
6	0/4	0/4	NS	0/1	0/4	0/4	NS	1/1	0/4	0/4	NS	0/1
7	0/4	0/4	0/4	0/1	1/4	1/4	0/4	0/1	0/4	0/4	0/4	0/1
10	0/4	0/4	NS	0/1	0/4	0/4	NS	0/1	0/4	1/4	NS	0/1
12	0/4	0/4	NS	0/1	0/4	0/4	NS	0/1	0/4	1/4	NS	0/1
14	0/4	0/4	0/4	0/1	0/4	0/4	0/4	0/1	0/4	0/4	0/4	0/1
18	0/4	0/4	NS	0/1	0/4	0/4	NS	0/1	0/4	0/4	NS	0/1
21	0/4	0/4	0/4	0/1	0/4	0/4	0/4	0/1	0/4	0/4	0/4	0/1

DPI days post-inoculation. N/O nasal/oral swab. R rectal swab. BC buffy coat. OF oral fluids. NS not sampled.

**Table 2:** Virus neutralization titers of virus challenged pigs and contacts.

Pig #	Group	3 dpi	7 dpi	14 dpi	21 dpi
344	IV	8	64	8	<8
345	IV	<8	32	32	8
346	IV	<8	64	16	8
347	IV	<8	32	8	8
348	IT	<8	<8	<8	<8
349	IT	<8	<8	<8	<8
350	IT	<8	<8	8	32
351	IT	<8	<8	16	8
352	IN	<8	<8	32	16
353	IN	<8	<8	<8	<8
354	IN	<8	<8	8	<8
355	IN	<8	<8	<8	<8
364	Contact	<8	<8	<8	<8
365	Contact	<8	<8	<8	<8
366	Contact	<8	<8	<8	<8

DPI days post-inoculation. IV intravenous. IT intratracheal. IN intranasal.

**Table 3:** rRT-PCR Ct values for select tissues of challenged pigs and contacts.

Pig #	Group	Nasal Turbinates	Tonsil	Mandibular LN	Tracheobronchial LN	Lung
344	IV	-	41.62	39.94	38.25	-
345	IV	-	-	-	-	-
346	IV	-	-	-	-	-
347	IV	-	-	-	-	-
348	IT	-	-	39.98	-	-
349	IT	-	-	-	-	-
350	IT	-	-	-	34.67	-
351	IT	-	-	-	-	-
352	IN	-	-	-	39.52	-
353	IN	-	-	-	-	-
354	IN	-	39.91	-	-	-
355	IN	-	-	-	-	-
364	Contact	-	-	39.55	-	-
365	Contact	-	-	-	-	-
366	Contact	-	-	-	-	-

“-“ = not detected. DPI days post-inoculation. IV intravenous. IT intratracheal. IN intranasal. LN lymph node.

## Data Availability

The data will be made available upon request via email to the corresponding authors.
